# 化学反应-顶空气相色谱法测定气相二氧化硅表面硅羟基

**DOI:** 10.3724/SP.J.1123.2020.11007

**Published:** 2021-07-08

**Authors:** Yun BAI, Xianjian DUAN, Dahai WANG, Guanghui HU, Chunlei WU, Mei ZHANG, Weili LIU

**Affiliations:** 1.北京市理化分析测试中心, 有机材料检测技术与质量评价北京市重点实验室, 北京 100089; 1. Beijing Key Laboratory of Detection Technology and Quality Evaluation of Organic Material, Beijing Centre for Physical and Chemical Analysis, Beijing 100089, China; 2.广州汇富研究院有限公司, 广东 广州 510663; 2. Guangzhou Huifu Research Institute Co., Ltd., Guangzhou 510663, China

**Keywords:** 顶空气相色谱, 化学反应, 硅羟基, 气相二氧化硅, 格氏试剂, 甲烷, headspace gas chromatography (HS-GC), chemical reaction, silanol group, fumed silica, Grignard reagent, methane

## Abstract

建立了一种基于化学反应-顶空气相色谱测定气相二氧化硅表面硅羟基含量的新方法。实验取气相二氧化硅放入顶空瓶中于105 ℃烘箱中加热2 h去除水分,将甲苯稀释的格氏试剂注入密闭的顶空瓶中,格氏试剂与气相二氧化硅表面硅羟基快速反应产生甲烷(CH_4_),甲烷量与气相二氧化硅表面硅羟基含量成正比。经过气相色谱-氢火焰离子化检测器测定甲烷,通过外标法定量,根据化学反应方程式计算出样品中羟基含量。同时对反应溶液用量与反应时间等条件进行优化,确定2.0 mL反应溶液,反应15 min为最优的前处理条件。结果表明,硅羟基含量与气相色谱信号值之间存在良好的线性相关性,相关系数为0.9990,相对标准偏差小于3%,本方法的检出限为0.30 mg/g,定量限为1.00 mg/g,开展了4家实验室对5个不同比表面积的样品测试,数据结果的重复性限(*r*)小于2.5%,再现性限(*R*)小于6.5%。该方法结合自动化技术,顶空反应操作简单,样品量和试剂用量少,准确性高,重复性好,优于酸碱滴定法,适用于快速检测气相二氧化硅表面硅羟基的含量,解决了硅羟基利用传统方法难以准确测定的难题。该方法的建立对我国二氧化硅产业硅羟基检测标准的制定和产业技术优化,均具有重要的理论和现实意义。

## Abstract (en)

Fumed silica is prepared by flame pyrolysis, where silicon halide is combusted in an oxygen-hydrogen flame, resulting in finely dispersed and thermally stable silicon dioxide. Because of its unique physical and chemical properties, including high porosity, large pore volumes, large specific area, and high chemical activity, fumed silica is widely used in rubbers, plastics, adhesives, paints, and printing inks for reinforcement, as well as in thixotropy, anti-setting, and anti-sagging applications. These functional properties of fumed silica are related to the silanol group on its surface. However, there is no accurate and convenient test method to determine the silanol group content on the surface of fumed silica. This work explores a novel method to determine the silanol group content on the surface of fumed silica by chemical reaction-headspace gas chromatography (HS-GC). Theoretically, by this method, the silanol group can rapidly react with the Grignard reagent and generate methane, the amount of which can be determined accurately by GC analysis. GC analysis was conducted using a headspace flask as a closed reactor to transform the silanol group into a volatile component through a chemical reaction, so as to realize the accurate determination of silica hydroxyl. The amount of methane produced in the reaction was directly proportional to the content of silanol groups on the surface of fumed silica. Therefore, the silanol group content was calculated using the chemical reaction equation. Before the experiment, fumed silica was dried for 2 h in an oven at 105 ℃ to remove adsorbed moisture. The dried fumed silica sample was then reacted with the Grignard reagent dispersed in toluene in an airtight reaction bottle. Toluene was used as a dispersion agent to promote contact and reaction between the fumed silica sample and Grignard reagent. The methane produced by the reaction was injected into a gas chromatograph for separation and further detected using a flame ionization detector (FID). Methane was quantified from the peak areas of the GC signals using the external standard method, and the silanol content in the sample was obtained. Simultaneously, factors influencing the outcome of the method, such as the dosage of the Grignard reagent and reaction time with it, were optimized by a comparison test. Accordingly, 2.0 mL of 0.3 mol/L Grignard reagent and a reaction time of 15 min were found to be optimal for testing. The test results showed that there was good linear correlation between the content of the silanol group and the GC signals, with a correlation coefficient of 0.9990. The limit of detection was 0.30 mg/g, and the limit of quantification was 1.00 mg/g. The relative standard deviation of reproducibility was less than 3%. Based on an interlaboratory test conducted by four laboratories on five samples with different silanol group contents, the repeatability limit (*r*) was less than 2.5%, and the reproducibility limit (*R*) was less than 6.5%. Compared with the traditional chemical method, the method involving HS-GC presents distinct advantages in terms of lower reagent consumption, high sensitivity, good stability, and reliability. It is suitable for the rapid detection of the silanol group content on the surface of fumed silica, and can aid in the quality control of fumed silica during its production and application. This method has important theoretical and practical significance for developing accurate methods to determine silica hydroxyl in the silicon industry for standards and the optimization of industrial technology. This study serves as a foundation to standardize and promote the rapid development of silicon material-related industries.

气相二氧化硅,俗称气相法白炭黑,是由卤硅烷在氢氧焰中经过高温水解制得轻质、高分散具有众多优质性能的无定形纳米二氧化硅^[1,2,3]^,广泛应用于工业填料^[4]^、催化剂^[5]^、药物载体^[6,7]^等。在这些应用中,气相二氧化硅表面特性对研究其应用的性能至关重要。由于硅羟基可以进行各种反应,如氯化、氨化、酯化等,因而控制气相二氧化硅表面羟基含量是非常重要的。硅羟基一般以孤立、相邻和双重等几种形式存在于气相二氧化硅表面,通常以孤立和相邻羟基为主^[8,9]^。气相二氧化硅表面的硅羟基极性强、活性高,使得其具有较强的亲水性,容易吸附水,且硅羟基表面能大,易于凝聚,导致气相二氧化硅在有机相中难以分散和浸润影响其应用。为了改善气相二氧化硅在有机溶剂中的分散性,需对其表面进行改性^[10]^。气相二氧化硅表面存在的硅羟基具有较好的补强、增稠和触变性能,但并非表面硅羟基含量越高越好,硅羟基含量越高,越容易导致硅橡胶在加工和储存过程中产生“结构化”效应;由于气相二氧化硅水分高,导致硅橡胶透明性下降、耐热性下降等。因此,准确测定气相二氧化硅表面硅羟基含量非常重要。

目前,硅羟基的分析方法主要有红外光谱法^[11]^、热重法^[12]^、化学滴定法^[13]^、核磁共振法^[14]^和气相色谱法^[15]^,其中,化学滴定法最为常用,其测试原理基于二氧化硅表面的硅羟基是路易斯酸,因而可以进行离子交换反应,通过这种离子交换记录采用酸碱滴定法可确定二氧化硅的硅羟基数量,进而计算得到二氧化硅表面硅羟基的含量;该方法操作简单易行,然而与气相二氧化硅的分散状态与时间、测试温度等因素有关,其对测试结果的影响比较大。气体测量法属于化学滴定法,是以在密闭体系反应的气体为基础的定量分析,采用格氏试剂或氢化铝锂与气相二氧化硅反应释放甲烷或者氢气表征测试硅羟基含量。但该法操作繁琐,误差较大,重复性不好,测试环境要求高,对水分敏感。李玉福等^[15]^研究用氢化铝锂-气相色谱法,采用气相色谱仪-热导池检测器,与化学方法相比,该法试剂与样品用量减少,检测灵敏度等方面明显提高,然而实验反应装置复杂,热导池检测器灵敏度差,重现性不佳,对于痕量物质的检测不适用,无法广泛应用。

顶空(HS)分析通常被定义为气相萃取,是对密闭容器中的液体或固体样品基质上方的气体进行定性和定量分析^[16,17]^。将顶空瓶作为一个密闭反应器,使气相二氧化硅表面硅羟基通过化学反应转化为挥发性组分,就可以进行气相色谱分析,从而实现硅羟基的准确测定^[18,19]^。

本研究借鉴了氢化铝锂-气相色谱法,提出了一种新方法:在顶空瓶中将硅羟基转化成CH_4_,反应产生的甲烷量与气相二氧化硅表面硅羟基含量成正比,通过测定反应所产生的甲烷气体的量计算气相二氧化硅表面硅羟基的含量。本文还考察了气相二氧化硅与稀释后的格氏试剂在顶空瓶中适宜的反应条件,开展了4家实验室间的比对验证试验。

## 1 实验部分

### 1.1 仪器、试剂与材料

顶空自动进样器(HS-20, SHIMADZU);气相色谱仪(GC-2010 Plus, SHIMADZU),配氢火焰离子化检测器;色谱柱:HP-MOLESIEVE毛细管柱(30 m×0.32 mm×25 μm);容量为1 L的带阀门聚四氟乙烯(PTFE)袋(E-Switch, 上海申源科学仪器有限公司);2.5 mL的注射器(江苏治宇医疗器材有限公司),用于量取格氏试剂、反应溶液;10 mL的气体注射器(Trajan Scientific Australia Pty Ltd)设有锁定阀,用于量取甲烷标气。

甲苯(色谱纯,纯度≥99.9%, Fisher Chemical);格式试剂(3.0 mol/L甲基碘化镁(CH_3_MgI乙醚溶液),JK Chemical);氢化钙(分析纯,国药集团化学试剂北京有限公司);甲烷气(纯度≥99.9%,北京海谱气体有限公司);高纯氮气(纯度≥99.999%,北京环宇京辉京城气体科技有限公司)。

5种不同比表面积气相二氧化硅购自湖北汇富纳米材料股份有限公司等企业。

### 1.2 操作条件

顶空条件:加热炉温度40 ℃,定量环温度50 ℃,传输线温度60 ℃,进样量1.0 mL,样品瓶平衡时间15 min。

气相色谱条件:柱箱温度50 ℃保持10 min,FID温度200 ℃,载气氮气,载气流速1.6 mL/min,分流比25:1。

### 1.3 标准样品的制备

取甲烷气体作为标准物质,使用注射器和气体收集袋以及适量的稀释气体(高纯氮气)制备6种体积分数分别为2.0%、5.0%、10.0%、30.0%、50.0%、100%的工作标准气体。分别取10 mL工作标准气体注入含有2.0 mL甲苯的密封顶空瓶中,以甲烷气在25 ℃、 1.01325×10^5^ Pa下的密度为0.6560 g/L计算,得0.131、0.328、0.656、1.968、3.280、6.560 mg甲烷气标准样品用于制作标准曲线。

### 1.4 样品前处理与制备

1.4.1 样品干燥减量

按照GB/T 5211.3-1985测试样品的干燥减量^[20]^。于105±2 ℃烘箱中干燥称量瓶和瓶盖2 h,取出放干燥器中冷却后,准确称量称量瓶和瓶盖质量*m*_1_,准确至1 mg;在称量瓶底部均匀铺放2 g左右样品,盖上瓶盖,准确称量质量*m*_2_,准确至1 mg;将称量瓶和样品在105±2 ℃烘箱中开盖干燥2 h;盖上瓶盖,放干燥器中冷却后,准确称取质量*m*_3_,准确至1 mg。

1.4.2 反应溶液配制

使用甲苯(实验前将氢化钙加入甲苯中除去其中的痕量水分)将格氏试剂稀释10倍。反应溶液按体积比在顶空瓶中现用现配,在最短的时间内用注射器完成所有样品瓶的注射。

1.4.3 空白样品

每批次样品测试前做空白试验。将2.0 mL反应溶液注射到密封的顶空瓶中制备3个平行的空白样品,与待测样品同样处理和测试。

1.4.4 样品制备

样品制备前,应先将样品瓶和瓶盖在烘箱中于105±2 ℃干燥2 h,取出放干燥器中冷却待用。在样品瓶中加入100 mg样品,准确称取样品质量*m*,准确至1 mg。在烘箱中于105±2 ℃下干燥2 h后,戴手套取出,立即盖上样品瓶盖,然后移至干燥器中冷却待用。

准确地将2.0 mL反应溶液注入样品瓶中,轻摇使样品粉末分散均匀,待测。

## 2 结果与讨论

### 2.1 硅羟基转化与色谱检测

格氏试剂与表面羟基中的活性氢反应,释放出CH_4_气体(反应式:SiOH+CH_3_MgI$\rightarrow$SiOMgI+CH_4_),过量的格氏试剂与硅羟基充分反应产生的甲烷气体,根据硅羟基的活泼氢来确定硅羟基含量是一个简捷有效的方法。

顶空瓶中释放出甲烷,经过顶空定量环取样和传输线传输,再由气相色谱仪测定甲烷,通过甲烷标准气体建立的校正曲线计算出该气相二氧化硅中硅羟基与格氏试剂反应生成的甲烷的量,然后根据反应式计算出气相二氧化硅中硅羟基的含量。本研究使用氮气作为气相色谱载气,顶空瓶中主要有甲烷和甲苯,经气相色谱分析,甲烷和甲苯的出峰时间分别为3.060 min和4.485 min。因此甲苯对甲烷的信号不会造成干扰,色谱图见图1。

**图 1:**
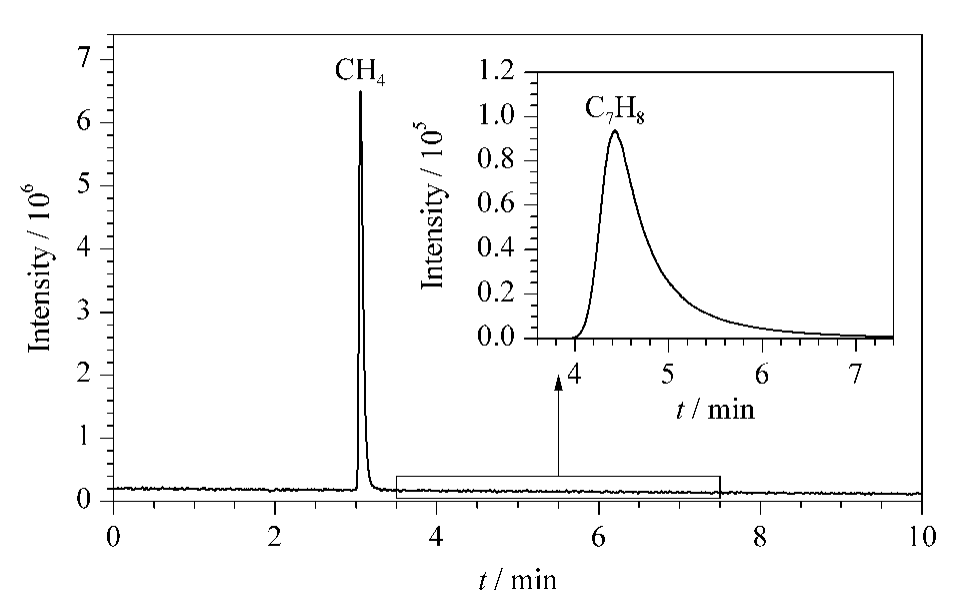
甲烷和甲苯的顶空气相色谱图

### 2.2 硅羟基含量的测定

2.2.1 定量计算

按1.4.1节对样品进行干燥减量处理,按公式(1)计算样品干燥减量的百分比*w*。

结合标准曲线外标法计算样品反应产生的甲烷质量(*X*, mg),通过对样品质量*m*(g)进行修正,按公式(2)计算气相二氧化硅表面的硅羟基含量(*α*_OH_, mg/g)。


(1) $w=\frac{m_{3}-m_{1}}{m_{2}-m_{1}} \times 100 \%$

(2) $\alpha_{\mathrm{OH}}=\frac{X}{m \times(1-w)} \times 1.060 2$

其中:1.0602为羟基与甲烷的相对分子质量比值。

2.2.2 方法优化

考察了反应溶液的加入量与反应时间两个重要因素的影响。若反应溶液加入量不足或者反应时间过短,反应不完全,导致测定结果偏小;若反应溶液加入量过多或者反应时间过长,会造成试剂的浪费或者仪器的损耗。因此,对反应溶液的加入量和反应时间进行优化。由图2可知,当反应溶液加入量在0.5~2.0 mL时,反应溶液的含量对实验结果有显著的影响;当反应溶液加入量大于2.0 mL时,甲烷峰面积的变化趋于平缓,说明反应达到平衡。因此,最佳的反应溶液加入量为2.0 mL。由图3可知,为了确保气相二氧化硅的硅羟基彻底转化为甲烷,最佳的平衡时间为15 min。

**图 2:**
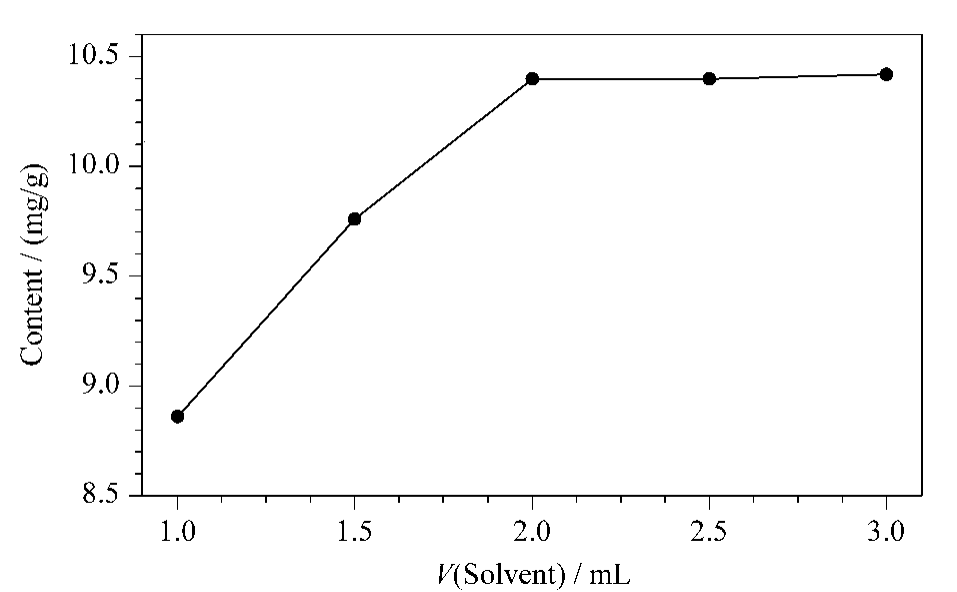
反应溶液用量对羟基含量的影响

**图 3:**
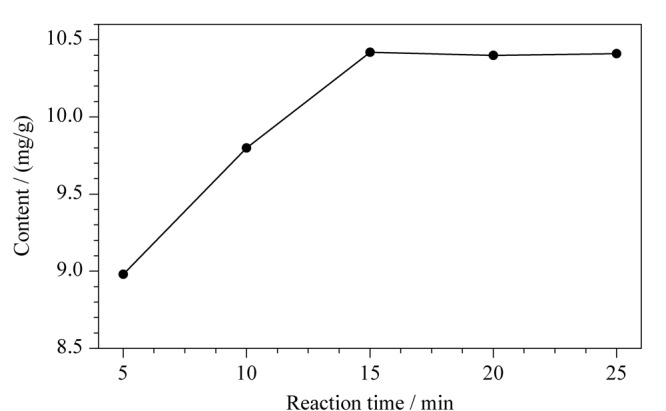
反应时间对羟基含量的影响

### 2.3 方法的线性关系、检出限和定量限

按1.3节配制不同浓度的甲烷标准样品进行色谱分析,以甲烷质量为横坐标,对应的峰面积为纵坐标建立标准工作曲线,得到甲烷的线性回归方程和相关系数。结果表明,甲烷质量在0.131~6.560 mg范围内线性关系良好*Y*=1.5×10^4^*X*+1×10^6^, 相关系数为0.9990。

参考IEC 62321-2008计算本方法的检出限(LOD)与定量限(LOQ)^[21]^。本方法检出限通过对空白样品进行6次重复、独立测定,由测定值的标准偏差乘以3得到,方法定量限以测定值的标准偏差乘以10确定;本方法硅羟基的检出限为0.30 mg/g,定量限为1.0 mg/g。

### 2.4 进样精密度和方法重复性

取0.656 mg的甲烷标准样品,按照1.2节仪器条件测定峰面积,重复测定6次,计算峰面积的RSD值,以此检验仪器条件的精密度。测定6份相同的气相二氧化硅样品,以生成的甲烷含量考察方法重复性,甲烷标准物质和样品产生的甲烷峰面积结果见表1。进样精密度RSD值为2.99%,方法重复性RSD值为2.12%,说明本方法具有良好的精密度。

**表 1:** 进样精密度和方法重复性(*n*=6)

Sample	Parallel experiment	Peak area	RSD/%
Methane	1	39801123	2.99
	2	37766276	
	3	39492892	
	4	37925226	
	5	40113784	
	6	37492892	
Fumed	1	54619818	2.12
silica	2	56479779	
	3	57535318	
	4	56350971	
	5	58001092	
	6	57252262	

### 2.5 方法比对

对3个不同硅羟基含量的气相二氧化硅样品,分别采用反应顶空-气相色谱法和酸碱滴定法进行测定,结果见表2。因气相二氧化硅表面硅羟基种类有孤立、邻位和孪生羟基,各自的活性不一样,酸碱滴定法基于表面硅羟基是路易斯酸的原理,假定样品在pH=4~9之间消耗的碱为硅羟基离解出的H^+^与碱反应,此pH值范围内,部分羟基不能离解出H^+^参与中和反应,测试结果偏低,另外酸碱滴定法测试时反应终点的确定受人为因素影响较大,因而其结果远远低于化学反应HS-GC的测试结果。说明化学反应HS-GC优于酸碱滴定法,可以更好地测定气相二氧化硅表面硅羟基的含量。

**表 2:** 化学反应HS-GC法与酸碱滴定法测定结果的比较

Sample No.	HS-GC/(mg/g)	Acid-base titration/(mg/g)
1	10.38	6.90
2	13.69	9.61
3	25.78	16.0

### 2.6 实验室间比对

运用本研究建立的方法,开展了4家实验室5个不同样品的比对验证,测试结果见表3。根据实验结果计算了实验室间的重复性限(*r*)和再现性限(*R*),实验室间*r*<2.44%,*R*<6.19%。结果见表4。表明该方法在不同实验室使用不同仪器时测定气相二氧化硅表面硅羟基含量具有良好的准确性。

**表 3:** 4个实验室中5个样品的硅羟基含量测定结果(*n*=3)

Lab	Sample 1	Sample 2	Sample 3	Sample 4	Sample 5
A	3.819	6.703	10.974	13.957	27.478
	3.698	7.249	10.319	14.680	26.970
	3.825	6.711	10.540	14.557	27.371
B	3.458	6.298	10.826	14.409	27.495
	3.431	6.330	10.902	14.300	26.752
	3.608	6.464	10.818	14.194	26.612
C	4.000	6.911	10.143	13.848	25.243
	3.982	7.002	9.866	14.043	24.853
	3.988	6.945	9.989	13.720	24.817
D	3.434	6.636	9.810	14.278	25.438
	3.590	6.308	10.330	14.022	25.775
	3.678	6.744	10.298	14.418	25.589

**表 4:** 实验室间的重复性限与再现性限测定结果

Parameter	Sample 1	Sample 2	Sample 3	Sample 4	Sample 5
Number of laboratories	4	4	4	4	4
Number of repeated determinations	3	3	3	3	3
Mean value/(mg/g)	3.71	6.69	10.40	14.20	26.20
Repeatability standard deviation/(mg/g)	0.07	0.16	0.16	0.19	0.25
r/%	1.89	2.44	1.83	1.37	0.96
Reproducibility standard deviation/(mg/g)	0.23	0.31	0.43	0.28	1.11
R/%	6.19	4.60	4.09	1.97	4.25

## 3 结论

本文基于格氏试剂与硅羟基快速反应,建立了化学反应型顶空与气相色谱联用技术测定气相二氧化硅表面硅羟基含量,并对稀释后的格氏试剂用量、反应时间进行了优化。所建立的方法具有较低的检出限,较高的精密度和准确度,通过开展方法比对和实验室间比对,进一步验证该方法准确可靠,为准确测定气相二氧化硅表面硅羟基含量提供了快速有效的方法,为促进气相二氧化硅及其下游产业的发展具有重要的意义。
